# Changes in Uterine Metabolome Associated with Metritis Development and Cure in Lactating Holstein Cows

**DOI:** 10.3390/metabo13111156

**Published:** 2023-11-16

**Authors:** Eduardo B. de Oliveira, Hugo F. Monteiro, Jessica M. V. Pereira, Deniece R. Williams, Richard V. Pereira, Noelia Silva Del Rio, Paulo R. Menta, Vinicius S. Machado, Fabio S. Lima

**Affiliations:** 1 Department of Population Health and Reproduction, School of Veterinary Medicine, University of California, Davis, CA 95106, USA; ebarrosdeoliveira@ucdavis.edu (E.B.d.O.); hmonteiro@ucdavis.edu (H.F.M.); rvpereira@ucdavis.edu (R.V.P.); nsilvadelrio@ucdavis.edu (N.S.D.R.); 2 Veterinary Medicine Teaching and Research Center, Tulare, CA 93274, USA; jmvpereira@uky.edu (J.M.V.P.); dvmwilliams@ucdavis.edu (D.R.W.); 3 Department of Animal Science, Federal University of Viçosa, Viçosa 36570-900, Brazil; 4 Department of Veterinary Sciences, Texas Tech University, Lubbock, TX 79409, USA; paulo.menta@ttu.edu (P.R.M.); vinicius.machado@ttu.edu (V.S.M.)

**Keywords:** metabolomics, uterine health, metritis, maleic acid, ornithine

## Abstract

The objective of this study was to identify alterations in the vaginal discharge (VD) metabolome and potential biomarkers to predict metritis development and a cure in dairy cows. This prospective cohort study was conducted on two dairies located in CA and TX. Vaginal discharge was evaluated and collected using the Metricheck^®^ device. Cows were examined for metritis at 4, 7, and 9 days in milk (DIM). Cows with a fetid, watery, and reddish-brown uterine discharge were classified as having metritis and randomized to receive ceftiofur (*n* = 10) or remain untreated (*n* = 7). A cure was defined as the absence of a fetid, watery, reddish-brown uterine discharge at 14 d after enrollment. Vaginal discharge samples were collected from 86 cows within 6 h after parturition, at 4 and 7 DIM, at metritis diagnosis, and at 4 and 7 days after metritis diagnosis. Cows with metritis (MET; *n* = 17) were paired with counterparts without metritis (HTH) of a similar DIM and parity (*n* = 34). The uterine metabolome was evaluated using untargeted gas chromatography time-of-flight mass spectrometry (GC-TOF-MS). Metabolomic data were analyzed using the MetaboAnalyst 5.0. Data were log-transformed and auto-scaled for normalization. Univariate analyses, including the fold-change, were performed to identify the metabolites linked to metritis development and its cure and principal component analysis and partial least squares discriminant analysis were performed to explain metabolite variance between animals developing or not developing metritis and being cured or not being cured of metritis. Comparing HTH with MET cows at calving, 12 metabolites were upregulated, and one was downregulated. At four and seven DIM, 51 and 74 metabolites, respectively, were altered between MET and HTH cows. After metritis development, three and five metabolites were upregulated in cows that were cured and in cows that received treatment and were cured, respectively. In all scenarios, the metabolites lignoceric, malic, and maleic acids, ornithine, and hypotaurine, which are associated with arginine/aminoacyl-tRNA biosynthesis and taurine/purine metabolism, were upregulated in HTH cows. Metritis was associated with changes in the uterine metabolome. Cows not being cured of metritis had changes in the uterus metabolome independent of receiving ceftiofur or remaining untreated. Metabolome analysis may be an important tool to understand the vaginal discharge changes during postpartum and the dynamics of metritis development and cures and help to identify biomarkers to predict metritis being cured.

## 1. Introduction

Metritis is a painful uterine pathology [1] that involves inflammation of all uterine layers (endometrium, myometrium, and serosa), and it is characterized by an abnormally enlarged uterus and a fetid, watery, red-brown uterine discharge within 21 days after parturition, with the incidence peaking within the first ten days postpartum [2]. Metritis has a high incidence (20–35%) in dairy cows [3] and has a marked negative impact on welfare, health, production, and reproduction [2,3,4]. Economic consequences for the individual animal and the herd range from U$212 to U$884 per case [5,6]. The disease is associated with signs of systemic illness (decreased milk yield, dullness, or other signs of toxemia), and approximately half of the metritic cows can have fever [rectal temperature (RT) ≥ 39.5 °C] [7].

Metritis is a complex multifactorial disease caused by a mixed bacterial infection [8,9]. Previous studies showed that the microbiota is identical between cows that develop metritis and healthy cows up until two days postpartum [10]. But after day two, the uterine microbiota diverges, and dysbiosis of the uterine microbiota is characterized by a loss of heterogeneity, a decrease in bacterial richness, and an increase in Bacteroidetes and Fusobacteria, particularly *Bacteroides*, Porphyromonas, and Fusobacterium, while the relative abundance of Proteobacteria and Tenericutes decreases [9].

During the transition into lactation, cows enter a negative energy balance, leading to body fat mobilization in the form of non-esterified fatty acids (NEFAs) and the accumulation of products of incomplete oxidation of NEFAs, such as beta-hydroxybutyrate (BHB) [11]. The decrease in glucose and calcium concentrations and the increase in NEFA and BHB concentrations are associated with immunosuppression and an increased risk of metritis [12]. A previous study reported that cows with metritis have alterations in metabolites related to carbohydrate metabolism, acute phase proteins, and proinflammatory cytokines at eight and four weeks before parturition [13]. In addition, products of bacterial metabolism, such as proteins, short-chain fatty acids, and other metabolites, affect immune functions [14]. In cows, the vascular degeneration that occurs shortly after calving allows blood to seep into the uterine lumen, which allows for the exchange of metabolites between blood and the uterine layers (endometrium, myometrium, and serosa) [15]. This exchange suggests that blood metabolites can affect the uterine microbiota, whilst microbial-derived metabolites may affect leukocytes in blood and tissues, and the crosstalk between metabolites of the host and pathogens plays a role in metritis development [16].

Studies have reported the associations of minerals or metabolites with immune function and metritis development [17,18]. Also, studies revealed that predictive models using cows’ sensor data [19] or machine learning algorithms [20] to predict a metritis cure might help improve the judicious use of antibiotics. However, studies characterizing the vaginal-uterine metabolome and potential biomarkers associated with the risk of metritis development and its cure remain scarce. Analytical approaches, such as metabolomics, are advancing and refer to analyzing concentration changes in small-molecule metabolites after organisms experience temporal and external stimuli [21]. The primary analytical approaches used in metabolomics rely on two techniques: nuclear magnetic resonance (NMR) and mass spectrometry (MS) [22]. The former determines the magnetic resonance of nuclei in the molecule and is suitable for detecting all compounds that contain hydrogen atoms, while the latter is the most frequently used platform in metabolomics quantification. Metabolomics improves the precision of identifying and quantifying low-molecular-weight metabolites and their intermediates in different biofluids or tissues, advancing the understanding of disease processes in dairy cows, as reported for milk fever, subclinical mastitis, and urine fingerprinting for metritis risk prediction [23,24,25].

Characterizing the vaginal-uterine metabolome during the peripartum can help unravel the crosstalk between host immunological factors and microbes that are key for metritis development and its cure and identify biomarkers that can help predictive models used to select cows that need to be treated for metritis. Therefore, the objectives of this study were to identify changes in the VD metabolome and biomarkers associated with metritis development, its cure, and antimicrobial use in Holstein cows using untargeted gas chromatography time-of-flight mass spectrometry. Our premise is that metritis development and its cure are associated with changes in the uterine metabolome after calving and that antimicrobial treatment is associated with uterine metabolome changes in metritic cows, and biomarkers that can be used for a metritis cure and its development exist.

## 2. Materials and Methods

### 2.1. Ethics and Animals

This prospective cohort study was conducted on two dairies in the Central Valley of California (4300 lactating Holstein cows) and one dairy in northwest Texas (2900 lactating Holstein cows) from August to October 2020, and January 2020 to May 2021, respectively. All herds milked only Holstein cows. The rolling herd average milk yield ranged from 10,150 to 12,000 kg. Postpartum pens had sand-bedded stalls and were equipped with sprinklers over the feeding areas activated when the environmental temperature rose above 21 °C. The postpartum diet was formulated to meet or exceed the dietary nutrient requirements for a lactating cow weighing 680 kg and producing 45 kg of 3.5% fat-corrected milk and 3.0% protein (NRC, 2001), and it was delivered as a TMR twice daily.

**Figure 1:**
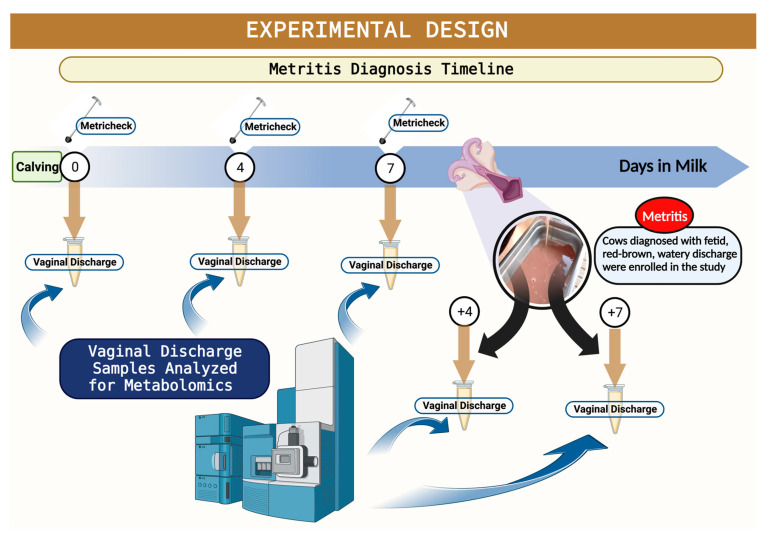
Schematic representation of the experimental design. Illustration of timeline for metritis diagnosis, metritis enrollment, and sampling for mass spectrophotometry assessment.

Considering that the incidence of metritis ranges from 20 to 35% in dairy cows [3] and we were expecting to have at least 15 cases of metritis, vaginal discharge samples were collected from 86 cows within 6 h after parturition, at 4 and 7 days in milk (DIM), at metritis diagnosis, and at 4 and 7 days after metritis diagnosis (Figure 1). All animals enrolled on the parturition day were assessed for metritis diagnosis at 5, 7, and 9 DIM, and vaginal discharge was evaluated using the Metricheck^®^ device (Simcro, Hamilton, Hamilton, New Zealand). Discharge retrieved from the vagina was scored as 1 = not fetid normal lochia, viscous, clear, red, or brown; 2 = cloudy mucoid discharge with flecks of pus; 3 = not fetid, mucopurulent discharge with <50% pus; 4 = not fetid mucopurulent white, yellow or reddish-brownish discharge with ≥50% pus; and, 5 = fetid, thin, serous, or watery, may have been reddish-brownish, with or without pieces of necrotic tissue present (adapted from Chenault et al., 2004 [26]). Cows with a fetid, watery, and reddish-brownish discharge, with or without pieces of necrotic tissue present, were classified as having metritis [27]. From the initial 86 cows from which the vaginal discharge was collected within 6 h after parturition, 17 animals developed metritis. Metritic cows were blocked by parity (primiparous and multiparous) and then randomly assigned to one of two treatments: (1) Ceftiofur [(*n* = 10) = subcutaneous injections of 6.6 mg/kg of ceftiofur crystalline-free acid (Excede^®^, Zoetis) in the base of the ear at D0 and D3. Live body weight was estimated using a heart girth measuring tape (Nasco Inc., Atkinson, WI, USA)]; (2) CON [(*n* = 7) = remained untreated at the time of metritis diagnosis. For vaginal discharge metabolomic analysis, cows with metritis (MET; *n* = 17) were paired with counterparts without metritis (HTH; *n* = 34) of a similar DIM and parity that originated from the initial 86 samples for the metabolomic analysis. Our rationale to use a 2:1 versus a 1:1 control-to-case ratio was that by selecting two counterparts without metritis who share similar days in milk and parity with the metritis group, we could enhance statistical efficiency by detecting smaller effect sizes or associations with greater precision, ultimately yielding more informative results. Moreover, the increased control-to-disease ratio enabled us to adjust more effectively for potential confounding variables, enhancing the overall robustness of our analysis. Additionally, the scarcity of research focusing on characterizing uterine metabolites in postpartum cows, especially in healthy individuals, underscores the importance of having a larger sample size for the healthy groups. This not only aids in better representing population dynamics for metritis but also reduces noise in the data. Cows with a vaginal discharge ≤4 were classified as HTH. Cows previously diagnosed with metritis with a vaginal discharge score <5 on d 14 after enrollment were considered cured. The post-enrollment exam on d 14 was performed by a veterinarian from the research team who was unaware of the treatment assignment. Cows within the withdrawal period for any antimicrobial or treated with any nonsteroidal and/or steroidal anti-inflammatory, cows submitted to caesarian section or fetotomy, or cows that aborted (<260 days of gestation) were not eligible for enrollment in the study.

### 2.2. Metabolomic Data Acquisition and Processing

Vaginal discharge was collected using the Metricheck^®^ device, transferred to two polypropylene vials, and stored at −80 °C until it was analyzed in an untargeted gas chromatography time-of-flight mass spectrometer (GC-TOF-MS) at the UC–Davis West Coast Metabolomics Center. The retention index and the complete mass spectrum were encoded as a string. All thresholds reflect the settings for ChromaTOF v. 4.0. Quantification was reported as the peak height using the unique ion as the default, unless a different quantification ion was manually set in the BinBase administration software version 1 BinView. We detected 174 known metabolites from 368 untargeted primary metabolites found in our analysis. A column of a 30 m length by 0.25 mm internal diameter with a 0.25 μm film made of 95% dimethyl/5diphenyl polysiloxanes was used in a Restek corporation Rtx-5Sil MS. The gas helium (99.99% purity) was used as a carrier for the analysis. The column temperature was set between 50 and 330 °C at a flow rate of 1 mL min^−1^. The oven temperature was set to 50 °C for 1 min, then ramped at 20 °C min^−1^ to 330 °C, and held constant for 5 min. Finally, the injection temperature was set to 50 °C and ramped to 250 °C by increments of 12 °C The retention of primary metabolites (amino acids, hydroxyl acids, carbohydrates, sugar acids, sterols, aromatics, nucleosides, amines, and various compounds) was evaluated.

### 2.3. Metabolomic Statistical Analysis

Metabolomic analyses were performed using Metaboanalyst 5.0 (www.metaboanalyst.ca (accessed multiple times in February 2022). Before data analysis, a data filtering and integrity check was performed to ensure that all the necessary information was collected (two classes, non-negative numbers for the compound concentration or peak intensity values, and missing value imputations). Data were log-transformed (base 10) and auto-scaled for normalization. For explanatory data analysis, a univariate analysis was performed. A fold-change analysis (FC) was performed with a threshold of 2 to identify candidate metabolites linked to metritis development and its cure. A volcano plot analysis was performed using a fold-change threshold (x) of 2 and *t*-test threshold (y) of 0.1 to select the essential features based on biological and statistical significance. Principal component analysis (PCA), partial-least square discriminant analysis (PLS-DA), and orthogonal PLS-DA analyses were performed to understand metabolite differences between animals developing or not developing metritis and being cured or not being cured of metritis. Pathways of different metabolites for models using two different organisms [cow (*Bos taurus*) and *E. coli*] were further screened using enrichment and topological analyses to identify the key pathway most highly correlated with metabolite differences.

## 3. Results

### 3.1. Number of Cows Enrolled Per Farm and Descriptive Data

Overall, 86 animals were enrolled, 44 in California and 42 in Texas. The number of animals developing metritis were nine and eight at the dairy in California and Texas, respectively. The average lactation number was 2.4. The mean DIM at metritis diagnosis was 6 days and the mean BCSs of animals enrolled and developing metritis were 3.2 and 3.5, respectively.

### 3.2. Changes in Uterine Metabolome in Cows Developing Metritis

**Figure 2:**
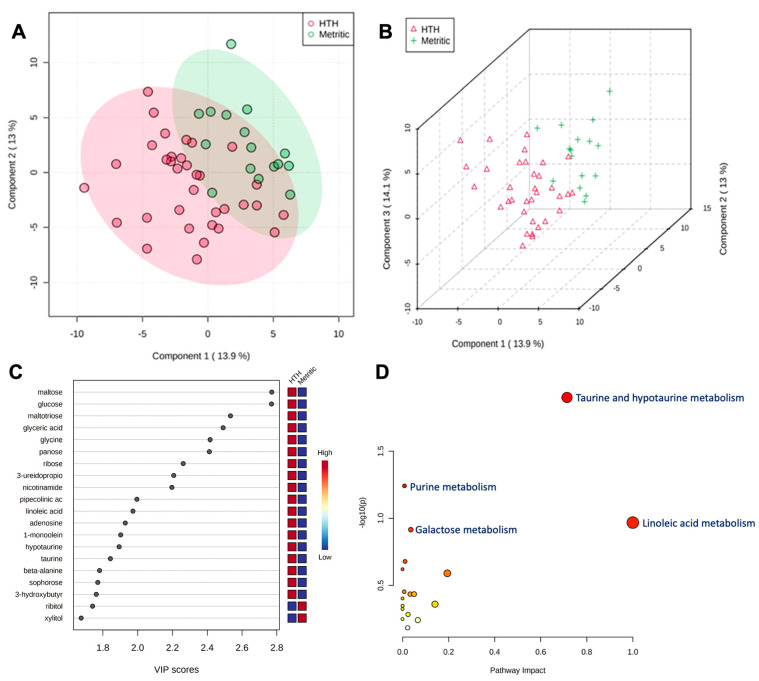
(**A**) Partial least square-discriminant analysis (PLS-DA) of vaginal-uterine discharge metabolites within 6 h postpartum in healthy cows compared to animals developing metritis. Score plot between the selected principal components; the explained variances are shown in brackets. (**B**) A 3-D representation of the known metabolome composition is displayed to demonstrate that there was a slight metabolome difference within 6 h postpartum between cows developing or not developing metritis. (**C**) Top 20 vaginal discharge metabolites detected via PLS-DA ranked by variable importance projection (VIP) scores. (**D**) Metabolic pathways associated with metritis at calving (d 0) based on enriched pathway analyses.

A total of 185 known and 236 unknown primary metabolites were identified in the vaginal discharge. Within six hours after calving, a comparison of metabolites revealed an association (adjusted *p* ≤ 0.05) with metritis development, with HTH vs. MET having 12 metabolites upregulated and one metabolite downregulated (Table 1). The PLS-DA with known metabolites confirmed the differences observed in the uterine metabolome, indicating a dispersion in the metabolite profile between HTH and metritic cows (Figure 2A,B). The top 20 uterine metabolites detected via PLS-DA ranked by variable importance projection (VIP) scores resembled most of the metabolites identified as different by the fold-change analysis (Figure 2C). Pathway analysis based on identified metabolites in cows not developing compared to cows developing metritis allowed for an understanding between the biological pathways for cows (*Bos taurus*) and *E. coli*. The subset of important metabolites was associated with glutathione, taurine, hypotaurine, alanine, aspartate, glutamate, D-glutamine, D-glutamate, and phenylalanine metabolism and aminoacyl-tRNA biosynthesis in *Bos taurus* organisms and associated with glutathione metabolism and aminoacyl-tRNA biosynthesis (Figure 2D).

**Figure 3:**
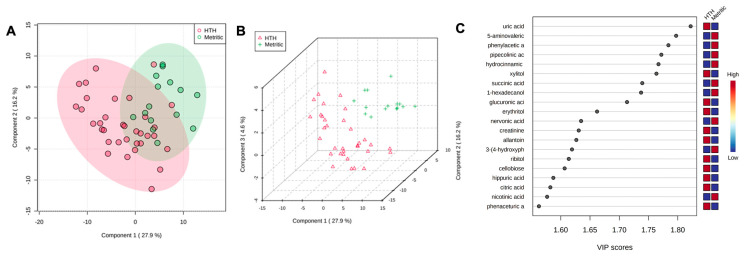
(**A**) Partial least square-discriminant analysis (PLS-DA) of vaginal-uterine discharge metabolites at 4 DIM in healthy cows compared to animals developing metritis. Score plot between the selected principal components; the explained variances are shown in brackets. (**B**) A 3-D representation of the known (**B**) metabolome composition is displayed to demonstrate that there is a metabolome difference at 4 DIM between cows developing or not developing metritis. (**C**) Top vaginal discharge metabolites detected via PLS-DA ranked based on variable importance projection (VIP) scores.

At 4 DIM, the fold-change analysis revealed that 51 metabolites were associated with metritis development (adjusted *p* ≤ 0.05). Comparing HTH to MET groups, 38 metabolites were upregulated, and 13 metabolites were downregulated. The top 50 metabolites are presented in Table 2. The PLS-DA with known metabolites at 4 DIM confirmed the differences observed in the uterine metabolome, indicating a dispersion in the metabolite profile between HTH and metritic cows (Figure 3A,B). The top 20 uterine metabolites detected via PLS-DA ranked by variable importance projection (VIP) scores resembled most of the metabolites identified as different based on the fold-change analysis (Figure 3C).

**Figure 4:**
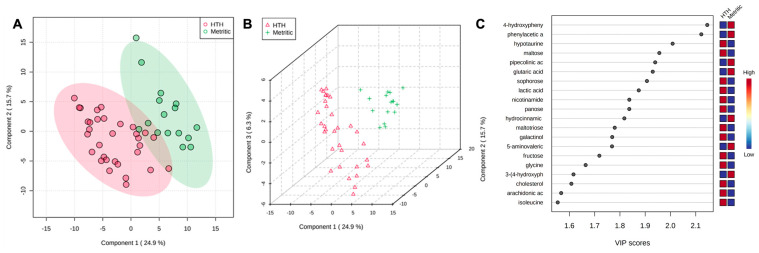
(**A**) Partial least square-discriminant analysis (PLS-DA) of vaginal-uterine discharge metabolites at 7 DIM in healthy cows compared to animals developing metritis. Score plot between the selected principal components; the explained variances are shown in brackets. (**B**) A 3-D representation of the known metabolome composition is displayed to demonstrate that there was a metabolome difference at 7 DIM between cows developing or not developing metritis. (**C**) Top vaginal discharge metabolites detected via PLS-DA and ranked based on variable importance projection (VIP) scores.

At 7 DIM, the fold-change analysis revealed that when comparing HTH to MET groups, 49 metabolites were upregulated, and 22 metabolites were downregulated. The top 50 metabolites are presented in Table 3. The PLS-DA with known metabolites at 7 DIM confirmed the differences observed in the uterine metabolome, indicating a dispersion in metabolite profile between HTH and metritic cows (Figure 4A,B). The top 20 uterine metabolites detected via PLS-DA and ranked by variable importance projection (VIP) scores resembled most of the metabolites identified as different based on the fold-change analysis (Figure 4C).

### 3.3. Changes in Uterine Metabolome in Cows Being Cured versus Cows Not Being Cured of Metritis

**Figure 5:**
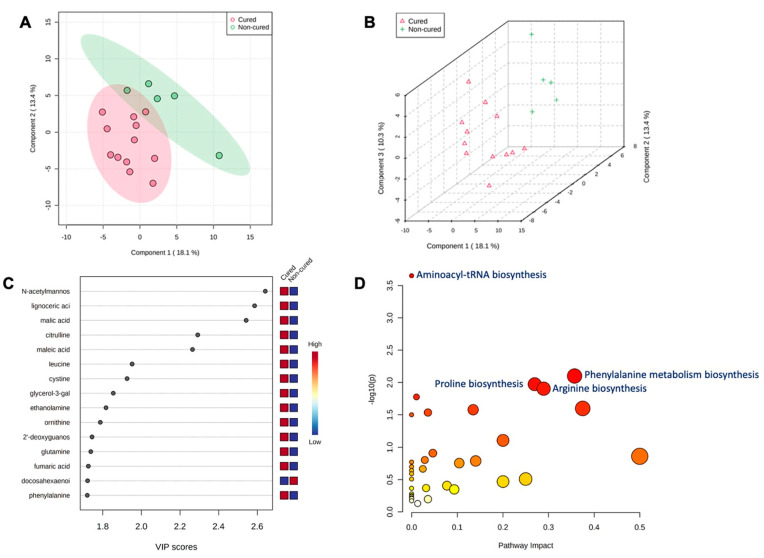
(**A**) Partial least square-discriminant analysis (PLS-DA) of vaginal-uterine discharge metabolites of metritic cows 4 days after metritis diagnosis. Score plot between the selected principal components; the explained variances are shown in brackets. (**B**) The 3-D representation of the known (**B**) metabolome composition demonstrated that 4 days after metritis diagnosis, there was a metabolome difference in cows being cured and not being cured of metritis. (**C**) Top vaginal discharge metabolites detected via PLS-DA and ranked based on variable importance projection (VIP) scores. (**D**) Metabolic pathways associated with metritis at calving (d 0) based on enriched pathway analyses.

After metritis development, three metabolites (lignoceric acid, maleic acid, and ornithine) were upregulated (*p* < 0.05) in cows that were cured compared to cows that were not cured of metritis and receiving or not receiving treatment. For the group of animals that received treatment and were cured, five metabolites (lignoceric acid, maleic acid, malic acid, N-acetylmannosamine, and ornithine) were upregulated (*p* < 0.05) compared to cows receiving treatment and not being cured of metritis. The PLS-DA with known metabolites confirmed the differences observed in the uterine metabolome, indicating a dispersion in the metabolite profile between cows being cured and not being cured of metritis (Figure 5A,B). The top 15 uterine metabolites detected via PLS-DA and ranked based on variable importance projection (VIP) scores represented the metabolites identified as being different based on the fold-change analysis (Figure 5C). Pathway analysis based on identified metabolites in cows being cured of metritis allowed for an understanding between the biological pathways for cows (*Bos taurus*) and *E. coli*. In both scenarios, the subset of essential metabolites was associated with arginine, proline, glyoxylate, dicarboxylate, and phenylalanine metabolism and aminoacyl-tRNA biosynthesis in *Bos taurus* organisms and associated with arginine and proline metabolism and arginine biosynthesis in *E. coli* organisms.

## 4. Discussion

As anticipated by our premise for the study, the characterization of primary metabolites depicted changes in the vaginal discharge metabolome in cows developing metritis that were dynamic over time and underscore differences between cows being cured and failing to be cured of metritis. Changes in metabolic pathways during the transition period, such as amino acid conversion to glucose and increased blood metabolites, such as NEFA and BHB, which are associated with dairy cow health and metritis, have been assessed in the literature throughout the years [11,28,29,30]. On the other hand, studies evaluating metabolomic changes in the uterus and related reproductive tract components during the first weeks postpartum and their association with metritis are only now emerging and need further exploration [16]. Our approach in the current study was supported by a premise that vaginal discharge, a sample that is relatively simpler and less invasive to collect than uterine fluid [16], could still be satisfactory to identify biomarkers for metritis development and its cure that could serve as data entry points to advance current predictive models for a metritis cure [18,20] and shed light on potential mechanisms of disease pathogenesis.

An interesting finding of this study was that the metabolome profile in the vaginal discharge changed from a few hours after calving up to the day of the metritis event, and the number of metabolites up- and downregulated increased over time up to 7 DIM within cows developing and not developing metritis. The leading metabolites up- and downregulated were sugar (e.g., glucose, maltose, panose), amino acid (adenosine, taurine, and hypotaurine)-derived metabolites, and other important organic compounds, such as nicotinamide and linoleic acid. A previous study suggested that preventing uterine disease in dairy cattle depends on avoiding, tolerating, and resisting pathogenic bacteria [31]. In other words, the concept proposed indicates that the prevention of metritis depends on the ability to mitigate tissue damage caused by pathogens through the neutralization of toxins, tissue repair, and immune competence. Important metabolites are known to have an essential role in the immune response and may represent the changes in uterus dysbiosis, toxin neutralization, and tissue repair [31,32,33].

The main pathways related to the metabolite changes in the uterus associated with the *Bos taurus* at an organism level are taurine, hypotaurine, purine, glutathione, and phenylalanine metabolism, which is positively associated with immunity and health. The nicotinamide concentration was increased in cows not developing metritis, suggesting that this metabolite regulates important physiologic processes in the uterus. Nicotinamide is a critical regulator maintaining important physiologic functions and controlling infection and inflammation [34]. Indeed, the essential redox effect of nicotinamide in promoting cellular oxidative (catabolic) metabolic disorders has been suggested and investigated as an anti-cancer and anti-aging therapeutic target [35]. Other metabolites being upregulated in cows not developing metritis were taurine and hypotaurine; both are antioxidant sulfur-containing amino acids that improve the immune function due to anti-apoptotic activities, antioxidant stress effects, and the regulation of mitochondrial function [36,37] Positive effects associated with these metabolites can be attributed to a potential improved immune response that may translate into the more rapid ability of the uterus tissue to respond to damage and consequently reduced the chances of pathogenic bacterial growth.

Cows have an established uterine microbiota within 20 min of calving [10]. The microbiota structure of a high proportion of proteobacteria is identical between cows that develop metritis and healthy cows up until two days postpartum, after which the bacterial community structure deviates in favor of a greater relative abundance of *Bacteroides*, Porphyromonas, and Fusobacterium in metritic cows [8,10,38]. Regarding the pathways associated with *E. coli* in the first week postpartum, it was mainly associated with specific pathways of glutathione, taurine, and hypotaurine metabolism and aminoacyl-tRNA biosynthesis, which are directly associated with protein biosynthesis, which induces translation and *E. coli* growth [39]. Glutathione, taurine, and hypotaurine metabolism pathways are associated with preventing inflammation and blocking bacterial growth, which could alleviate bacterial dysbiosis [40].

There is a scarcity of studies investigating the uterine postpartum metabolome either for health cows or for cows with the occurrence and progression of metritis. On the other hand, the individual metabolites reported as upregulated in the current study and their action on metabolic pathways have been discussed in other species. An in vitro study investigating the effect of L-arginine on human endometrial cell proliferation reported that supraphysiological concentrations of L-arginine led to increased endometrial RL95-2 cell proliferation and reduced apoptosis, which had a positive impact on endometrial epithelium regeneration, growth, and health [41]. Another relevant aspect of the arginine biosynthesis pathway is the broad role of L-arginine as a precursor of important molecules for cellular physiology, such as nitric oxide and its conversion to ornithine via arginase [42]. In this study, we observed that ornithine was one of the metabolites upregulated in cows not developing metritis. Studies investigating the effect of the abundance of arginine and ornithine in pigs reported a positive effect on the uterus, placental–fetal blood flow, fetoplacental nutrition, and endometrial epithelial cell proliferation [43]. Additionally, some studies highlighted possible connections between L-arginine and *Bacteroides* spp. and found that an increased arginine concentration correlated with reduced *Bacteroides* growth. Metritic cows had a greater abundance of total bacteria and a greater abundance of *Bacteroides pyogenes* (*B. pyogenes*), *Porphyromonas levii* (*P. levii*), and *Fusobacterium necrophorum* (*F. necrophorum*) [15]. A possible interpretation to reconcile these studies is that metabolites in the vaginal discharge of cows developing metritis, even after disease establishment, can alter pathogenic growth and reduce dysbiosis. In that case, it could reduce the severity and improve curing independent of treatment, as shown in the present study.

The upregulation of antioxidant amino acids, such as lignoceric, malic, maleic, and hypotaurine in healthy cows was an important finding in this study. Those amino acids are associated with taurine and purine metabolism and have been reported as important compounds in cows’ immune functions [44,45]. In addition to the antioxidant effect, they regulate mitochondrial protein synthesis by enhancing electron transport chain activity and controlling apoptosis due to the regulation of excessive superoxide generation [39]. Cows not being cured of metritis had significant changes in the uterus metabolome independent of receiving ceftiofur or remaining untreated. Cow not being cured had a lower concentration of lignoceric, malic, maleic, and lauric acids and ornithine. The main pathways related to the metabolite changes in the vaginal discharge microbiome associated with the *Bos taurus* at an organism level were aminoacyl-tRNA biosynthesis, glutathione, taurine, and hypotaurine metabolism, which match with the pathways associated with cows that did not develop metritis and suggest that these pathways are associated with immunity, uterus integrity, and pathogenic bacteria reductions, which may reduce the severity of the disease and improve healing from metritis. The main pathways related to the metabolite changes in the uterus associated with *E. coli* at the organism level were arginine biosynthesis and arginine and proline metabolism pathways reported as associated with gut microecology and *E. coli* reductions [40].

One unique aspect of the current study was that we collected vaginal discharge for the metabolome analysis instead of uterine fluid [16], which in itself is a source of variation that can limit the comparison with other studies. However, as acknowledged previously, the vaginal discharge is a less invasive and simpler method to retrieve samples that, unless a cow has severe vaginitis after calving, should mainly represent the contents being released by the enlarged postpartum uterus that remains involuted with an open cervix. The Metricheck tool used has been compared with other standard methods for evaluations of vaginal discharge for the diagnosis of uterine disease, such as cytology, manual evaluations, and vaginoscopy, and it was found to have higher sensitivity than vaginoscopy for the diagnosis of uterine diseases, such endometritis [46,47]. Further studies should focus on increasing the sample size of animals with metritis and assessing the metabolites associated with a spontaneous cure. In this study, we could not analyze metabolite changes in cows being self-cured of metritis due to the limited number of animals left untreated and being cured. Additionally, performing studies using different methods for discharge collection and comparing how the metabolome from plasma and uterine fluid is associated will be essential to determine what metabolites are related to immune functions and pathogenic bacterial proliferation in the uterus. Although the new approaches can be beneficial in identifying key biomarkers associated with an improved immune function, reduced pathogenic bacterial growth in the uterus, and improved uterine health, the current finding already offers a list of potential biomarkers that integrated predictive models for metritis could advance.

## 5. Conclusions

In summary, untargeted GC-TOF-MS metabolomic analysis of the vaginal discharge of cows in the first week postpartum, at metritis diagnosis, and at 4 and 7 days after metritis diagnosis, highlighted changes in the uterine metabolome in the first week postpartum in cows developing metritis compared to healthy animals. For the metritic group, there were significant changes in the uterine metabolome associated with a cure. In all scenarios, the metabolites lignoceric, malic, and maleic acids, ornithine, and hypotaurine, which are associated with arginine/aminoacyl-tRNA biosynthesis and taurine/purine metabolism, were upregulated in the HTH group and in cows being cured of metritis. Also, cows not being cured of metritis had significant changes in the uterus metabolome independent of receiving ceftiofur or remaining untreated. Metabolome analysis may be an important tool to understand changes in the uterus during the postpartum period and the dynamics of metritis development.

## Figures and Tables

**Table 1:** Important metabolites that were up- and downregulated within 6 h postpartum in healthy cows compared to animals developing metritis.

Compounds	FC ^1^	Log2(FC)	Raw.pval	−log10(p)
1 Maltose 2 Nicotinamide 3 Maltotriose 4 1,5-anhydroglucitol 5 Panose 6 Glucose 7 Adenosine 8 Linoleic acid 9 Alpha-aminoadipic acid 10 Taurine 11 Hypotaurine 12 Inosine 13 Thymine	8.2008 2.2286 8.2341 2.1199 6.5977 4.0647 2.0373 2.6882 3.9039 2.6779 2.0738 2.5176 0.38734	3.0358 1.1562 3.0416 1.084 2.722 2.0231 1.0266 1.4266 1.9649 1.4211 1.0523 1.332 −1.3683	0.001909 0.0027633 0.0072505 0.012228 0.013923 0.016681 0.021417 0.02161 0.022083 0.026722 0.055062 0.078835 0.080836	2.7192 2.5586 2.1396 1.9127 1.8563 1.7778 1.6692 1.6653 1.6559 1.5731 1.2591 1.1033 1.0924

**^1^** FC: fold-change analysis with a threshold of 2.

**Table 2:** Metabolites that were up- and downregulated at 4 DIM in healthy cows compared to animals developing metritis.

Compounds	t.stat	*p*-Value	−log10(p)	FDR ^1^
1 Allantoin 2 Hypotaurine 3 Uric acid 4 1-hexadecanol 5 Cellobiose 6 Glucuronic acid 7 Erythritol 8 5-aminovaleric acid 9 Pipecolinic acid 10 Creatinine 11 Citric acid 12 Lactic acid 13 3-ureidopropionate 14 Xylitol 15 Allantoic acid 16 Isothreonic acid 17 Phenylacetic acid 18 Nicotinamide 19 4-hydroxyphenylacetic acid 20 Hippuric acid 21 Nervonic acid 22 Cadaverine 23 Glycine 24 Glycocyamine 25 Succinic acid 26 Pseudo uridine 27 Putrescine 28 D-erythro-sphingosine 29 Ribitol 30 Phenaceturic acid 31 Oxoproline 32 Xylulose 33 Arabitol 34 Cysteine 35 Ribose 36 Threonic acid 37 3-(4-hydroxyphenyl) propionic acid 38 Hydrocinnamic acid 39 Maltose 40 Catechol 41 P-cresol 42 Sorbitol 43 Glycolic acid 44 Octadecanol 45 Behenic acid 46 Glyceric acid 47 Maltotriose 48 2-hydroxybutanoic acid 49 2-deoxypentotol 50 Tyrosine	4.4416 4.252 4.1915 −4.1581 4.1178 4.0816 4.0113 −3.951 −3.9048 3.8466 3.8016 3.755 3.7381 3.6841 3.6706 3.6278 −3.6003 3.5962 −3.5726 3.5171 −3.452 −3.4318 3.4276 3.4252 −3.3832 3.3478 −3.2783 −3.2286 3.2244 3.2082 3.1472 3.114 3.1024 3.0958 3.0776 3.0125 −2.9573 −2.9345 2.9032 2.8957 2.8655 2.8519 −2.8469 −2.7999 −2.767 2.6815 2.6716 2.6675 2.6377 2.5708	15.3971 × 10^−5^ 9.9863 × 10^−5^ 0.00012129 0.00013494 0.00015347 0.00017218 0.00021499 0.00025986 0.0003002 0.00035973 0.00041339 0.00047699 0.0005024 0.00059224 0.00061711 0.00070238 0.00076317 0.00077266 0.00082918 0.00097869 0.001187 0.0012597 0.0012756 0.0012847 0.001 4529 0.0016107 0.0019694 0.0022705 0.0022982 0.0024068 0.0028608 0.0031399 0.0032436 0.0033043 0.0034762 0.0041638 0.004844 0.005154 0.00561 0.0057249 0.0062093 0.0064385 0.0065247 0.0073937 0.0080657 0.010078 0.01034 0.01045 0.011282 0.013372	4.2678 4.0006 3.9162 3.8699 3.814 3.764 3.6676 3.5853 3.5226 3.444 3.3836 3.3215 3.2989 3.2275 3.2096 3.1534 3.1174 3.112 3.0813 3.0094 2.9255 2.8997 2.8943 2.8912 2.8378 2.793 2.7057 2.6439 2.6386 2.6186 2.5435 2.5031 2.489 2.4809 2.4589 2.3805 2.3148 2.2879 2.251 2.2422 2.207 2.1912 2.1854 2.1311 2.0934 1.9966 1.9855 1.9809 1.9476 1.8738	0.0049933 0.0049933 0.0049933 0.0049933 0.0049933 0.0049933 0.0053441 0.0056519 0.0058039 0.0062594 0.0065391 0.0067244 0.0067244 0.0071585 0.0071585 0.007469 0.007469 0.007469 0.0075936 0.0085146 0.0093138 0.0093138 0.0093138 0.0093138 0.010112 0.010779 0.012692 0.013789 0.013789 0.01396 0.016058 0.01691 0.01691 0.01691 0.017282 0.020125 0.02278 0.0236 0.024903 0.024903 0.026352 0.026402 0.026402 0.029239 0.031187 0.037881 0.037881 0.037881 0.040064 0.046533

Important features selected based on a volcano plot with a fold-change (FC) threshold (x) of 2 and *t*-test threshold (y) of 0.1. Both fold-changes and *p*-values were log-transformed. The further its position is away from the (0,0), the more significant the feature is. The position is referring to the combination of the log-transformed *p*-value ((-log10(p) (*y* axis) and log 2-fold-change (*x* axis)). ^1^ FDR: adjusted *p*-values from the *t*-tests.

**Table 3:** Important metabolites that were up- and downregulated at 7 DIM in healthy cows compared to animals developing metritis.

Compounds	t.stat	*p*-Value	−log10(p)	FDR ^1^
1 4-hydroxyphenylacetic acid 2 Phenylacetic acid 3 Hypotaurine 4 Maltose 5 Pipecolinic acid 6 Sophorose 7 Nicotinamide 8 Glutaric acid 9 Galactinol 10 Panose 11 5-aminovaleric acid 12 Lactic acid 13 Fructose 14 Maltotriose 15 Hydrocinnamic acid 16 2-hydroxybutanoic acid 17 Glycine 18 3-(4-hydroxyphenyl) propionic acid 19 Piperidone 20 Taurine 21 Arachidonic acid 22 Oxoproline 23 3-ureidopropionate 24 Lactinol 25 Cholesterol 26 Cystine 27 Glucose 28 Nervonic acid 29 Xylose 30 Creatinine 31 1,2-anhydro-myo-inositol 32 Myo-inositol 33 Serine 34 Citric acid 35 Isoleucine 36 Glutamic acid 37 Cellobiose 38 1-hexadecanol 39 Threonic acid 40 1-monoolein 41 Uric acid 42 Oleic acid 43 Ribose 44 Cysteine 45 Nicotinic acid 46 Linoleic acid 47 D-erythro-sphingosine 48 Thymidine 49 Pseudo uridine 50 Isothreonic acid	−7.6977 −7.2237 6.479 6.281 −6.2315 6.2077 5.9226 −5.5935 5.3672 5.282 −5.2647 5.2319 5.0786 4.9746 −4.9171 4.3955 4.2499 4.1443 4.024 4.0158 3.9972 3.9167 3.8288 3.7716 3.761 3.7455 3.7383 −3.6912 −3.5946 3.588 3.5793 3.562 −3.4475 3.4404 3.3787 3.3418 3.3172 −3.312 3.2696 3.246 3.2416 −3.236 3.2332 3.165 −3.1618 3.116 −3.061 −3.0488 3.0166 2.9366	8.2798 × 10^−10^ 4.2118 × 10^−9^ 5.5259 × 10^−8^ 1.0965 × 10^−7^ 1.3013 × 10^−7^ 1.4129 × 10^−7^ 3.7817 × 10^−7^ 1.1722 × 10^−6^ 2.5392 × 10^−6^ 3.3924 × 10^−6^ 3.5981 × 10^−6^ 4.0207 × 10^−6^ 6.75 × 10^−6^ 9.5721 × 10^−6^ 1.1603 × 10^−5^ 6.4644 × 10^−5^ 0.00010331 0.00014462 0.00021137 0.00021689 0.00022992 0.00029544 0.00038759 0.00046193 0.00047709 0.00050011 0.00051127 0.00058986 0.00078901 0.00080456 0.0008259 0.00086949 0.00122 0.0012457 0.0014918 0.0016601 0.0017826 0.0018093 0.0020431 0.0021857 0.0022137 0.0022489 0.0022673 0.0027498 0.0027745 0.0031546 0.0036766 0.0038021 0.0041551 0.0051679	9.082 8.3755 7.2576 6.96 6.8856 6.8499 6.4223 5.931 5.5953 5.4695 5.4439 5.3957 5.1707 5.019 4.9354 4.1895 3.9859 3.8398 3.6749 3.6638 3.6384 3.5295 3.4116 3.3354 3.3214 3.3009 3.2914 3.2292 3.1029 3.0944 3.0831 3.0607 2.9136 2.9046 2.8263 2.7799 2.749 2.7425 2.6897 2.6604 2.6549 2.648 2.6445 2.5607 2.5568 2.5011 2.4346 2.42 2.3814 2.2867	1.4407 × 10^−7^ 3.6643 × 10^−7^ 3.205 × 10^−6^ 4.0975 × 10^−6^ 4.0975 × 10^−6^ 4.0975 × 10^−6^ 9.4003 × 10^−6^ 2.5495 × 10^−5^ 4.9091 × 10^−5^ 5.6915 × 10^−5^ 5.6915 × 10^−5^ 5.83 × 10^−5^ 9.0346 × 10^−5^ 0.00011897 0.00013459 0.000703 0.0010574 0.001398 0.001887 0.001887 0.001905 0.0023367 0.0029322 0.0032948 0.0032948 0.0032948 0.0032948 0.0036656 0.0046357 0.0046357 0.0046357 0.0047279 0.006375 0.006375 0.0074162 0.0080237 0.0082846 0.0082846 0.0091154 0.0091746 0.0091746 0.0091746 0.0091746 0.010728 0.010728 0.011933 0.013611 0.013783 0.014755 0.017984

Important features selected based on a volcano plot with a fold-change (FC) threshold (x) of 2 and *t*-test threshold (y) of 0.1. Both fold-changes and *p*-values were log transformed. The further its position away from the (0,0), the more significant the feature is. The position is referring to the combination of the log-transformed *p*-value ((-log10(p) (*y* axis) and log 2-fold-change (*x* axis)). ^1^ FDR: adjusted *p*-values from the *t*-tests.

## Data Availability

The original contributions presented in the study are included in the. article, further inquiries can be directed to the corresponding author.
